# Functional properties and sequence variation of HTLV-1 p13

**DOI:** 10.1186/s12977-020-00517-1

**Published:** 2020-05-12

**Authors:** Maria Omsland, Micol Silic-Benussi, Ramona Moles, Sarkis Sarkis, Damian F. J. Purcell, David Yurick, Georges Khoury, Donna M. D’Agostino, Vincenzo Ciminale, Genoveffa Franchini

**Affiliations:** 1 Animal Models and Retroviral Vaccines Section, Vaccine Branch, Center for Cancer Research, National Cancer Institute, National Institutes of Health, Bethesda, MD USA; 2 Veneto Institute of Oncology IOV-IRCCS, Padua, Italy; 3 Department of Microbiology and Immunology, The Peter Doherty Institute for Infection and Immunity, University of Melbourne, Parkville, VIC Australia; 4 Department of Biomedical Sciences, University of Padua, Padua, Italy; 5 Department of Surgery, Oncology, and Gastroenterology, University of Padua, Padua, Italy; 6 Present Address: Division of Microbiology and Immunology, Yerkes National Primate Research Center, Emory University, Atlanta, GA USA

**Keywords:** Human T cell leukemia virus type-1, HTLV-1, HTLV-1C, p13, Mitochondria, Cell death

## Abstract

Human T cell leukemia virus type-1 (HTLV-1) was the first retrovirus found to cause cancer in humans, but the mechanisms that drive the development of leukemia and other diseases associated with HTLV-1 infection remain to be fully understood. This review describes the functional properties of p13, an 87-amino acid protein coded by HTLV-1 open reading frame II (*orf*-*II*). p13 is mainly localized in the inner membrane of the mitochondria, where it induces potassium (K^+^) influx and reactive oxygen species (ROS) production, which can trigger either proliferation or apoptosis, depending on the ROS setpoint of the cell. Recent evidence indicates that p13 may influence the cell’s innate immune response to viral infection and the infected cell phenotype. Association of the HTLV-1 transcriptional activator, Tax, with p13 increases p13’s stability, leads to its partial co-localization with Tax in nuclear speckles, and reduces the ability of Tax to interact with the transcription cofactor CBP/p300. Comparison of p13 sequences isolated from HTLV-1-infected individuals revealed a small number of amino acid variations in the domains controlling the subcellular localization of the protein. Disruptive mutations of p13 were found in samples obtained from asymptomatic patients with low proviral load. p13 sequences of HTLV-1 subtype C isolates from indigenous Australian patients showed a high degree of identity among each other, with all samples containing a pattern of 5 amino acids that distinguished them from other subtypes. Further characterization of p13’s functional properties and sequence variants may lead to a deeper understanding of the impact of p13 as a contributor to the clinical manifestations of HTLV-1 infection.

## Background

Human T cell leukemia virus type 1 (HTLV-1) is a retrovirus that causes an aggressive neoplasm of mature CD4^+^ T cells called adult T cell leukemia (ATL), as well as a variety of inflammatory diseases including uveitis, infectious dermatitis, Sjögren’s syndrome, and HTLV-1-associated myelopathy/tropical spastic paraparesis (HAM/TSP) [1–8]. Although at least 5–10 million people are infected with HTLV-1 worldwide, only a small percentage will develop clinically relevant symptoms [9]. The factors that determine the clinical outcome of HTLV-1 infection are not fully understood, but studies aimed at investigating the viral determinant of HTLV-1 pathogenicity have underscored the oncogenic potential of Tax and HBZ [10].

Despite having a relatively small genome of approximately 9000 nt [11], HTLV-1 expresses multiple gene products through the transcription of both strands of its proviral genome, complex mRNA splicing, and ribosomal frameshifting, resulting in the production of protease and polymerase enzymes, the structural Gag and Env proteins, and the non-structural proteins Tax, Rex, p21Rex, p30, p13, p12/p8, and HBZ. This review describes the biological properties of p13, a small protein encoded by the second open reading frame of the X region (*orf*-*II*) [12]. As we explore here, recent studies have revealed interesting variations in p13 sequences isolated from different patients infected with HTLV-1 [13, 14] that may possibly be relevant to the life cycle and pathogenic properties of HTLV-1.

### Expression and intracellular localization of p13

**Fig. 1:**
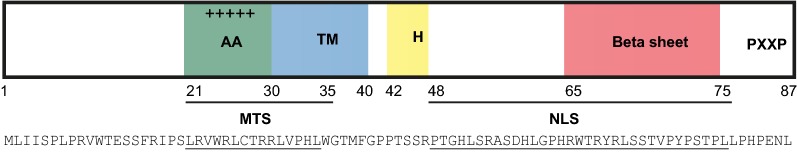
p13 domain structure. Schematic representation of the domain structure of p13. AA indicates the amphipathic α-helix overlapping with the mitochondrial targeting signal (MTS, amino acids 21–35) and +++ indicates the four arginines present in the MTS. The transmembrane region (TM) includes amino acids 30–40. A region with a high flexibility score (H) spans amino acids 42–48. A predicted β-sheet structure spans amino acids 65–75. The proline-rich C-terminus contains two overlapping P-x-x-P motifs implicated in interactions with SH3 domain-containing proteins. A putative cryptic nuclear localization sequence (NLS) is mapped to a region spanning residues 43–80. This figure was adapted from Figure 1 in [87]

Early studies of *orf*-*II* revealed that it codes for two proteins: p30, a 241-residue nuclear/nucleolar protein expressed from a doubly-spliced mRNA, and p13, an 87-residue protein coded by a singly-spliced mRNA corresponding to the carboxy-terminal portion of p30 [12, 15, 16]. Analyses of the expression kinetics of the viral transcripts in infected cells showed that the p13 and p30 mRNAs accumulate late in the replication cycle, together with mRNAs encoding structural proteins [17, 18]. Initial studies carried out in a HeLa-derived cell line transiently transfected with p13 expression plasmids indicated that the protein accumulated in punctate structures located in the cytosol and perinuclear area, and in the nucleus but not nucleoli [15]. Subsequent co-localization analysis with compartment markers revealed that the punctate structures containing p13 were in fact mitochondria [19]. The results of mutational analyses and assays with GFP-tagged portions of p13 led to the identification of the minimal mitochondrial targeting signal (MTS) that is necessary and sufficient to determine the protein’s mitochondrial accumulation (Fig. 1) [19]. Circular dichroism analysis showed that the p13 MTS folds into an amphipathic alpha helical structure containing four arginines [20]. Unlike canonical MTS, the p13 MTS differs is not located at the amino terminus of the protein, it is not cleaved upon import, and it does not require the presence of the four arginines for mitochondrial localization [20]. The mitochondrial localization of p13 was confirmed by confocal microscopy analysis and a combination of electron microscopy and biochemical fractionation studies, which revealed that p13 is mainly inserted in the inner mitochondrial membrane [20]. Confocal microscopy analysis also confirmed mitochondrial localization in transfected primary rat embryo fibroblasts and the T cell acute lymphoblastic leukemia (T-ALL) cell line Jurkat [21].

**Fig. 2:**
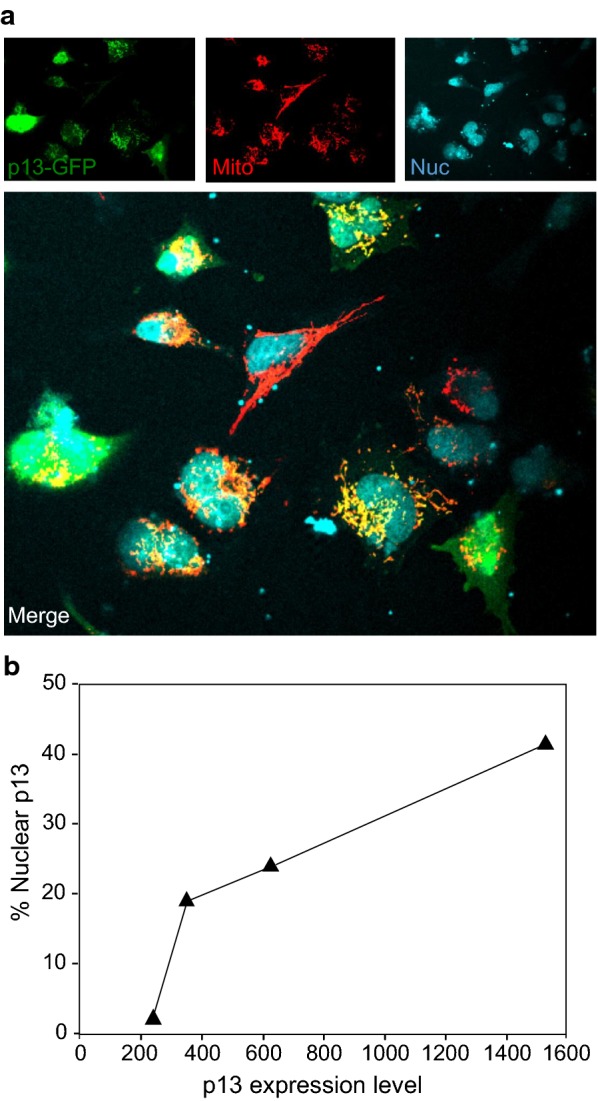
Intracellular localization of p13-GFP. **a** Confocal microscopy analysis of HeLa cells transfected with a p13-GFP-expressing plasmid and labelled with an antibody recognizing the mitochondrial protein HSP60 (Santa Cruz Biotechnology) and an Alexa-546-conjugated secondary antibody (Life Technologies). Nuclei were visualized using Vibrant DyeCycle Ruby (Life Technologies). Nuclei, mitochondria, and p13 are shown in blue, red, and green, respectively. **b** Quantitative analysis of the percentage of p13-GFP signal detected in nucleus in relation to the total p13-GFP signal measured in the same cell

The MTS of p13 acts as a dominant targeting signal that is necessary for the mitochondrial accumulation of p13, and is sufficient to direct the mitochondrial accumulation of heterologous proteins such as GFP [19]. The 13-kDa size of p13 is well below the cut-off of the nuclear pore, suggesting that the protein should be able to freely diffuse in and out of the nucleus. As depicted in Fig. 1, p13 is believed to contain a nuclear localization signal (NLS) positioned after its MTS. The existence of this NLS was inferred from observations from a series of deletion mutants of p30 fused to GFP [22], and further analysis is required to verify its impact on p13’s intracellular compartmentalization. A study by Andresen et al. showed that p13 becomes more stable when co-expressed with Tax, that it is modified by ubiquitination, and that a small fraction of p13 is localized to nuclear speckles containing Tax and SC35 (Fig. 2) [23]. Interestingly, the nuclear localization of p13 was more prominent when the protein was fused to green fluorescent protein (GFP) or to the hemagglutinin (HA) epitope tag (unpublished data). The nuclear accumulation of p13-GFP also appeared to be proportional to the expression levels of the protein (Fig. 2). These findings suggest that p13 may accumulate in the nucleus when a certain concentration threshold is reached, which might be favored by Tax or the presence of tags such as ubiquitin.

### Functional properties of p13

#### Effects of p13 on K^+^ flux

**Fig. 3:**
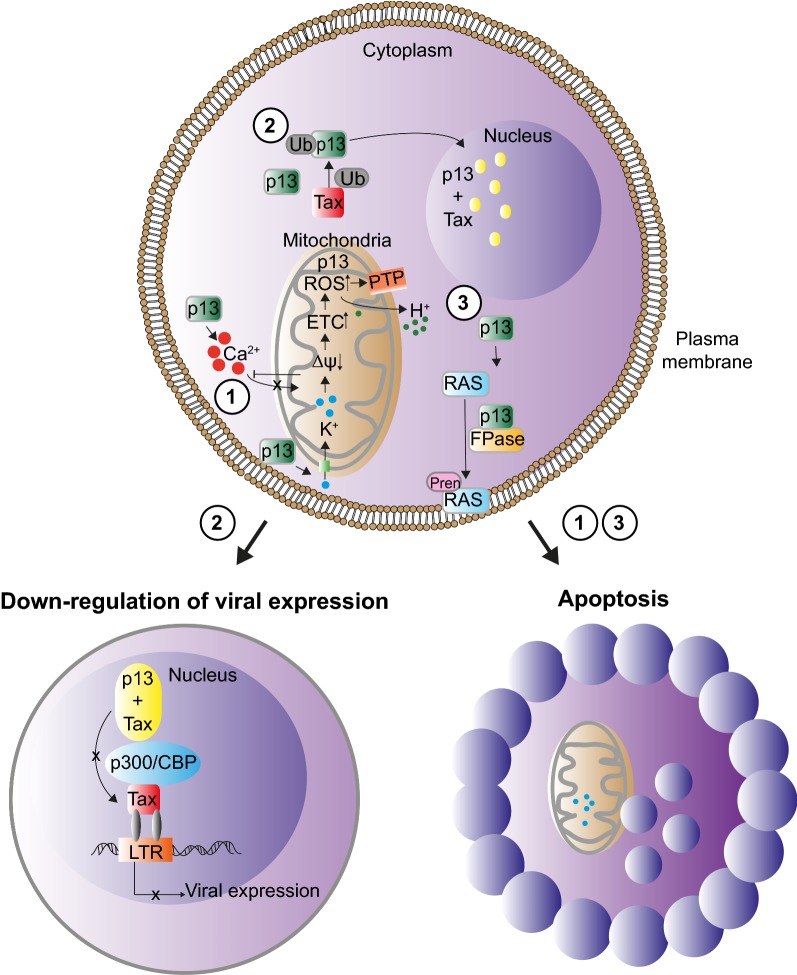
Overview of p13 biology. (1) p13 accumulates in the inner mitochondrial membrane. It induces an inward K^+^ current which leads to decreased mitochondrial membrane potential (Δψ) and a compensatory increase in the activity of the ETC, resulting in increased ROS production, and opening of the PTP, leading to mitochondrial swelling and apoptosis. p13 also interferes with the uptake of Ca^2+^ by mitochondria, which leads to an accumulation of cytosolic calcium. (2) When p13 is co-expressed with Tax, p13 becomes ubiquitinated and more stable. The protein partially accumulates in the nucleus, where p13 and Tax co-localize in nuclear speckles. (3) Interaction of p13 with farnesyl pyrophosphate synthase (FPase) interferes with Ras prenylation and targeting to the plasma membrane

Studies carried out using synthetic p13 and purified mitochondria showed that the protein induces a potassium influx through the inner mitochondrial membrane (Δψ) [20, 24]. The entry of K^+^ increases the activity of the electron transport chain (ETC), which extrudes a higher number of H^+^ and thus balances the entry of K^+^ positive charges. Although this ramping up of the ETC maintains the potential of the mitochondrial membrane, it also favors the production of reactive oxygen species (ROS). Increased ROS may trigger the opening of the mitochondrial permeability transition pore (PTP), an event that can lead to cell death [25] (Fig. 3).

Cells expressing p13 have fragmented mitochondria [20]. The effect of p13 in both isolated mitochondria and in cells is dose-dependent: When p13 is low, the entry of K^+^ is counterbalanced by increased ETC activity. At higher levels of p13, cells show mitochondrial depolarization and substantial fragmentation. Although the four arginines in the amphipathic alpha helix are not essential for mitochondrial targeting, they are required to induce the inflow of K^+^ into mitochondria and for mitochondrial fragmentation [20, 24].

#### p13 and mitochondrial ROS

ROS are powerful second messengers that control multiple signal transduction pathways. Depending on their levels, ROS may favor cell proliferation, neoplastic transformation, or cell death. The effects of ROS in cell turnover have been compared to a rheostat [26]: a moderate increase in ROS stimulates healthy resting cells to proliferate, and a further increase will favor tumor transformation. Excessive ROS production will lead to DNA damage and apoptotic cell death.

In agreement with observations made in isolated mitochondria, p13 was found to increase ROS production in several cell models (e.g. HeLa cells, primary T cells, and Jurkat T-ALL cells) [25]. These findings suggest that, in the context of the viral life cycle, p13 might contribute to an expansion of the pool of infected T cells, but trigger apoptosis of infected cells that acquire a transformed phenotype. In line with this model, the expression of p13 has been shown to lead to the activation of primary T cells and increased apoptotic death of Jurkat T-ALL cells [25].

#### Role of p13 in calcium signaling

p13 also influences the mitochondrial uptake of calcium ions. In response to physiological stimuli (nutrients, hormones, neurotransmitters), Ca^2+^ rapidly accumulates in the mitochondrial matrix, where it stimulates enzymes of oxidative metabolism to produce ATP [27]. In contrast, mitochondrial calcium overload triggers sustained PTP opening, which leads to cell death [28].

Experiments carried out using Ca^2+^-sensitive aequorin probes localized to different cellular compartments (mitochondria, ER, or cytosol) showed that p13 inhibits the influx of Ca^2+^ into mitochondria, resulting in a local increase in the cytosolic concentration of Ca^2+^ (Fig. 3) [29]. This effect is interesting in light of the crucial role of Ca^2+^ signaling in the activation and survival of T cells, the primary targets of HTLV-1 infection. It would be interesting to determine if p13 functionally interacts with p12, a small, hydrophobic *orf*-*I* protein that localizes in the ER, binds calreticulin, and increases cytosolic Ca^2+^ levels, resulting in the activation of nuclear factor of activated T cells (NFAT) [15, 30].

#### Effects of p13 on cell signaling pathways

Lefebvre et al. showed that p13 negatively influences the Ras signal transduction pathway by binding to farnesyl pyrophosphate synthase (FPase), a key enzyme in the synthesis of substrates necessary for the prenylation of Ras and its association with the plasma membrane (Fig. 2) [31]. This property may contribute to the ability of p13 to suppress the transformation of rat embryo fibroblasts driven by the combination of Ras and Myc [32]. p13 also inhibits the proliferation of Jurkat cells when grown at high culture density and favors cell death under glucose deprivation or upon treatment with C2-ceramide, an inducer of the intrinsic pathway of apoptosis [33].

The C-terminal portion of p13 (and p30) is rich in prolines, including those arranged in a sequence resembling P-x-x-P (PPII) helical motifs that interact with SH3 domain-containing proteins such as the Src family kinases (SFKs). Using in vitro binding and kinase assays, Tibaldi et al. confirmed that p13 is able to associate with SFKs Src, Fyn, Fgr, and Lyn [34]. This interaction led to increased kinase activity, but the effect was blocked in the presence of a p13 peptide spanning residues 61–87. Further analysis of the p13-Lyn interaction provided evidence that p13 is able to direct Lyn to mitochondria, resulting in an accumulation of p13-Lyn complexes in the intermembrane space instead of the inner membrane location observed for p13 alone. This change in p13’s submitochondrial localization was accompanied by an attenuation in the p13-mediated loss of mitochondrial membrane potential [34]. These p13-SFK interactions share some similarities with properties described for HIV-1 Nef, whose interactions with the Hck, Lyn, and Src SFKs play a positive role in viral replication [35, 36].

The role of autophagy in recycling damaged or unneeded organelles has gained increased attention in recent years. Mitophagy is a mitochondria-specific subtype of this recycling mechanism that can be induced by different factors, including viral infection [37]. Mitophagy induced by Hepatitis viruses B and C has been suggested to reduce apoptosis and increase viral persistence [38, 39]. Coxsackie B virus (CVB) uses the mitophagy process for its viral spread [40], and classical swine fever virus (CSFV) induces mitophagy to inhibit apoptosis [41]. The effects of p13 on mitochondrial depolarization, swelling, and general homeostasis might therefore trigger the mitophagy process. The role of p13 in regulating essential parts of the mitophagy pathway would be an interesting point of investigation for future studies.

#### Impact of p13 on Tax function

Studies on the interplay between p13 and Tax [23] indicated that p13 binds directly to Tax and interferes with its ability to associate with CBP/p300, an interaction that is needed for Tax-mediated activation of transcription from the long terminal repeat (LTR) promoter. Together with its late expression kinetics, this property supports a role for p13 as an ‘off’ switch that favors entrance into a latent state of infection, which is likely important for virus persistence in the host [23].

### p13-like proteins in other deltaretroviruses

HTLV-1 is classified in the deltaretrovirus genus, together with the related HTLV-2, 3, and 4 orthologues that infect nonhuman primates as simian T cell leukemia virus (STLVs); and bovine leukemia virus (BLV). Due to their shared molecular features, the human and simian viruses are collectively referred to as primate T-lymphotropic viruses (PTLVs).

HTLV-2 circulates as 2 major subtypes, A and B, and is present mainly in South American indigenous populations, western and central Africa, and among intravenous drug users [42]. The pathogenicity of HTLV-2 is not clearly defined [43], but infection with this virus appears to significantly increase all-cause mortality [44]. The HTLV-2 x-II *orf* codes for a protein named p28 that, despite limited sequence identity, shares several functional properties with HTLV-1 p30 [45]. Transfection assays carried out using a plasmid coding for an HTLV-2A x-II *orf* carrying an epitope tag at the 3ʹ end did not reveal expression of any proteins smaller than p28, suggesting that HTLV-2A does not code for a p13 homologue [46]. Analogous experiments have not been carried out for HTLV-2B.

HTLV-3 [47, 48] and HTLV-4 [49] were identified in individuals from Cameroonian rainforests and are of unknown pathogenicity [50]. As part of a detailed analysis of the coding potential of HTLV-4 isolate 1863LE, Switzer et al. identified an open reading frame (*orf*-*IV*) that codes for a 68-amino acid protein with 75% similarity to portions of p13 located after the MTS and transmembrane motif [49]. Interestingly, alignment of this predicted protein with p13, HTLV-2 p28, and related *orfs* in STLV-2 (*orf*-*II*) and HTLV-3 (*orf*-*III*) revealed a highly conserved stretch of amino acids corresponding to p13 residues 59–84 in all of the sequences [49]. Afonso et al. [51] recently described a divergent STLV-1 isolate that contains mutations that affect splicing and/or codons that disrupt both the x-II and x-I *orfs*. An extension of this analysis to other STLVs and HTLVs showed disruption of either or both of these *orfs* in several other PTLVs [51].

BLV is a widespread pathogen that infects cattle, water buffalo, and zebus, and causes B-lymphocytosis, leukemia, and lymphoma. Accessory proteins coded by BLV include G4, a 105-amino acid protein that is targeted to mitochondria, and to a lesser extent, to the nucleus [31]. p13 and G4 share very little sequence identity (26.4%, unpublished data), and its mitochondrial targeting depends on both an amino-proximal hydrophobic alpha-helix and a more carboxy-terminal arginine-rich amphipathic alpha helix [31]. In contrast to the tumor suppressor-like property observed for p13, BLV G4 cooperates with H-Ras in a rat embryo fibroblast transformation model, and is needed for BLV-driven tumor development in a sheep model [52].

### p13 and the host immune response

Several studies over the past decade have revealed important roles for mitochondria in immune responses [53]. Many viruses encode mitochondrial proteins that are important for viral spread and persistence [54–56], and several viruses target MAVS (mitochondrial antiviral-signaling protein), a cellular protein localized in the outer mitochondrial membrane, mitochondrial-associated membranes, and peroxisomes that plays a critical role in innate immune response against RNA viruses [57–59]. The ability of HTLV-1 to infect monocytes suggests that the virus might impinge on the host’s innate immune responses [60–63].

One way that p13 might impact the host’s immune response could be through its regulation of cell death. Cell death mechanisms induced through mitochondrial pathways are known to enable the release of circular mitochondrial DNA (mtDNA) from mitochondria to the cytosol [64]. The presence of mtDNA in the cytosol can further trigger inflammatory responses in the host through the cyclic GMP–AMP synthase (cGAS)-stimulator of interferon genes (STING) signaling pathway [65, 66]. The possible impact of p13 on anti-viral innate immunity is currently under investigation in our laboratories. The size and shape of mitochondria are controlled by the balance between mitochondrial fusion and fission [67]. This dynamic is intimately connected to the pattern of differentiation and activation of cells involved in immune responses. Naive effector T cells have small, round mitochondria, and their activation to effector T cells increases mitochondrial fission [68]. Mitochondrial fusion is prevalent in memory T cells, and results in elongated, tubular mitochondria and higher mitochondrial mass [68]. In their initial description of alternatively spliced HTLV-1 mRNAs, Berneman et al. showed that the p13 mRNA was expressed in 6 out of 10 ATLL samples, but was not detected in 3 PBMC samples from healthy HTLV-1 carriers [69]. It would be interesting to investigate if p13 might be involved in the selection of memory T-cells, which may represent the cell of origin of ATL [70].

#### In vivo studies of p13

In vivo studies performed in rabbits using the molecular clone ACH.1 or a derivative containing a mutation that abolished expression of both *orf*-*II* proteins (ACH.30/13.1) showed that animals inoculated with ACH.30/13.1 had lower proviral loads than animals inoculated with ACH.1. These results suggest that p13 and/or p30 might be essential for the maintenance of viral loads in vivo [71]. The importance of p13 in vivo was confirmed in a more recent study of a molecular clone lacking the p13 initiation codon but still able to produce p30 [72]. However, in both studies the p13 knock-out was obtained by substituting its ATG start codon with GAT, which also disrupts the HBZ *orf* coded on the antisense strand [73]. New studies that do not interrupt the HBZ *orf* are thus needed to verify the importance of p13 alone.

### Sequence variation of p13

While the HTLV-1 genome structure and sequence are in general highly conserved, sequence variations in HTLV-1 LTR segments are used to classify HTLV-1 isolates into 7 molecular subtypes with characteristic geographic distributions: subtypes A (further divided into 5 subgroups), B, C, D, E, F, and G [9]. Several studies have been directed at identifying subtypes and variants that might correlate with the clinical characteristics of infected patients. A study by Nozuma et al. comparing isolates from asymptomatic carriers and HAM/TSP patients confirmed the virus’s high degree of sequence conservation but also provided evidence for an association between HAM/TSP and infection with the “transcontinental” HTLV-1A subgroup [14]. Interestingly, this subgroup also showed a significantly higher frequency of mutations, mainly single nucleotide substitutions [14]. Kuramitsu investigated viral parameters associated with indeterminate HTLV-1 western blot (WB) results, defined as a lack of antibody reactivity against gp46Env and/or Gag proteins p53, p24 and p19, and observed that WB-indeterminate patients had a higher frequency of viral sequence variations compared to patients with positive WB results [13]. Even more notable was the median proviral load, which was almost 100-fold lower in this group. Kuramitsu et al. suggest that the low proviral load might be due to mutations in the provirus that reduce viral replication and limit dissemination within the host [13].

**Fig. 4:**
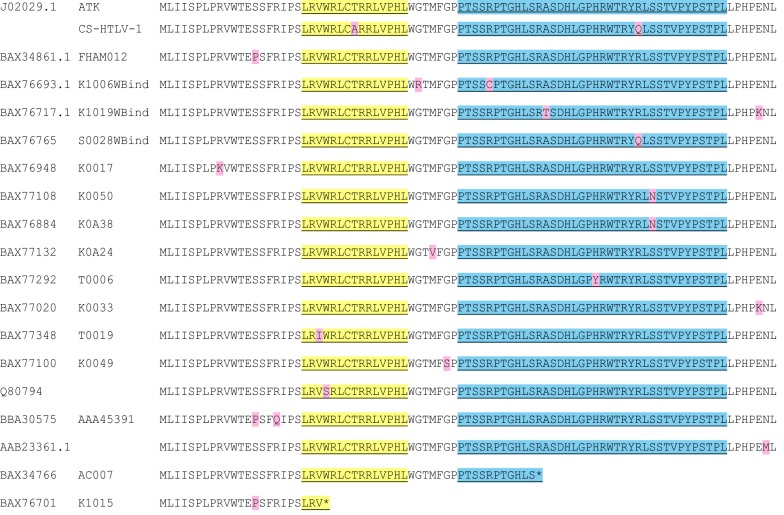
HTLV-1A p13 sequence alignments. GenBank accession numbers and isolate names are indicated in the left column. Isolate CS-HTLV [75] is not deposited in GenBank. Its p13 sequence was determined by Sanger sequencing in our laboratory. Sequence variations are highlighted in pink. The mitochondrial targeting signal (MTS) and the putative nuclear localization sequence (NLS) are highlighted in yellow and blue, respectively. The multi-alignment was performed with the Mega7 program using default parameters

Using the datasets from these studies, we analyzed the p13 sequence in isolates from patients with different clinical characteristics. As a reference sequence, we used the p13 sequence coded by viral isolate ATK (accession number J02029.1), which was derived from a Japanese ATL patient and is considered to be a prototype for subtype A viruses [11]. A search using the Basic Local Alignment Search Tool (BLAST; https://blast.ncbi.nlm.nih.gov/Blast.cgi) yielded 69 hits obtained from published sequences [12–14, 74]. Although the majority (78%) of the sequences shared 100% identity with p13 coded by ATK (not shown), 17 isolates showed amino acid variations (Fig. 4). Interestingly, two of these sequences have stop codons resulting in the early termination of p13 (isolates K1015 and AC0007, Fig. 4). K1015, an isolate from the WB-indeterminate group [13], has an early termination codon at position 24 that results in the loss of all the domains essential for p13’s intracellular targeting and functional properties. This isolate also has stop codons in Pol and Env (not shown). Isolate AC0007, from an asymptomatic carrier [14], has a stop codon at position 54 and thus retains the MTS and transmembrane sequence, but lacks most of the putative nuclear localization sequence (NLS) of p13 (Fig. 4). Two other isolates have a single amino acid difference in the MTS domain. None of the sequences in the BLAST search matched with p13 coded by the CS-clone of HTLV-1, which was derived from a North American ATL patient [75] and carries a single amino acid difference in the MTS and one in the putative NLS. All of the samples with variations in the MTS were from HAM/TSP or ATL patients who had positive WB results. None of the variations in the MTS involve the four arginines identified as critical for p13 function [20].

Amino acid variations within p13’s putative NLS were identified in six of the sequences: three in the WB-indeterminate group, and three in the WB-positive group (Fig. 4) [13]. Two of the latter sequences carried an asparagine in place of serine at position 70 (S70N). Although Kuramitsu et al. concluded that the samples from the group with indeterminate WB results had the highest frequency of sequence variations [13], the majority of isolates with p13 variations were from patients with either an HTLV-1 associated disease or detectable antigens in WB analysis. Further studies are needed to determine if the variations observed in the patients with positive WB results or disease actually influence the function of p13. It is notable that the two isolates coding for truncated p13 proteins were obtained from asymptomatic carriers who did not have positive WB results, suggesting a possible link between p13 and disease development.

### Sequence variations in p13 coded by HTLV-1 subtypes A and C

HTLV-1 C, present mainly in Australia and Melanesia, has recently gained attention due to it high prevalence (in up to 40% of adults) in indigenous populations living in remote regions of central Australia [76]. Unlike subtype A, subtype C seems to be more frequently associated with inflammatory disease in the lungs, causing bronchiectasis or bronchitis with high morbidity [77, 78]. However, cases of infectious dermatitis and ATL have also been reported for HTLV-1C [79, 80]. The reasons for the different pathogenic properties of the two subtypes are still unclear.

**Fig. 5:**
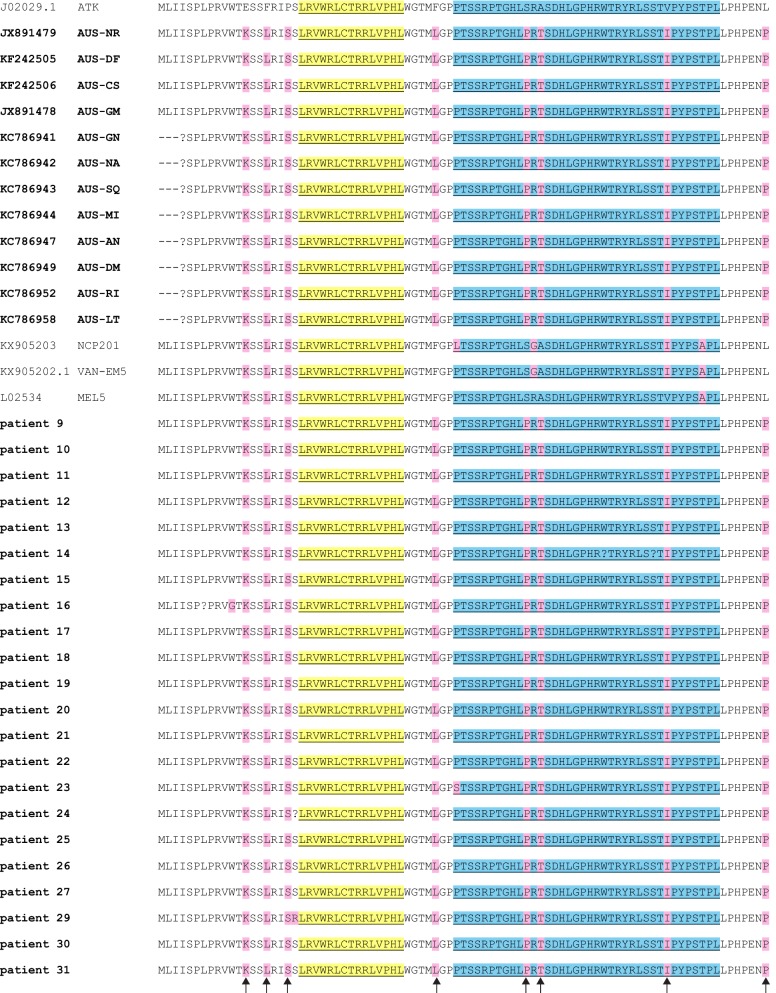
Alignments of p13 coded by HTLV-1A prototype ATK and 37 HTLV-1C isolates. GenBank accession numbers and/or isolate names are indicated in the left column. Patient isolates 9–31 are described in [82]. Isolates from Australia are indicated in bold. Isolates NCP201, VAN-EM5, and MEL5 were obtained from New Caledonia, Vanuatu [84], and the Solomon Islands [85], respectively. Sequence variations are highlighted in pink. The mitochondrial targeting signal (MTS) and the putative nuclear localization sequence (NLS) are highlighted in yellow and blue, respectively. Arrows at the bottom of the figure indicate an 8-residue ‘signature’ present in all Australian isolates (see text). The multi-alignment was performed with the Mega7 program using default parameters

Pairwise sequence comparisons at the nucleotide and amino acid levels between HTLV-1A and HTLV-1C show that the structural genes, *gag*, *pol*, *pro*, and *env* are more highly conserved compared to the pX region, suggesting that the X-region proteins might contribute to the different clinical manifestations observed in infected individuals [81]. To gain additional insight into the role of p13 in HTLV-1 pathogenesis, we extended our analysis to the p13 proteins coded by 37 subtype C sequences, 34 of which were isolated from Australian patients (Fig. 5) [82–85]. Sequence information for 4 amino-terminal amino acids was not available for 8 GenBank entries (prefix KC in Fig. 5). Since these amino acids were invariant in the other sequences, we chose to compare amino acids 5–87 for the full set of sequences. The analysis revealed 89–95% identity between ATK and the 37 HTLV-1C sequences, 90–100% identity among all HTLV-1C sequences, and 98–100% identity among the 34 Australian sequences, with 31 out of 34 showing 100% identity. Interestingly, all 34 Australian sequences share an 8-amino acid ‘signature’ that distinguishes them from ATK. The first three residues of this signature are present in the entire set of HTLV-1C sequences (Fig. 5).

Overall, this analysis confirms similar amino acid sequences and conservation of the principal domains described for HTLV-1A p13, including the MTS, the transmembrane region, the putative NLS region and the P-x-x-P SH3 binding motif. It is notable that the MTS is perfectly matched among all of the sequences. Interestingly, three of the ‘Australian signature’ residues are located in the putative NLS region, and the last signature residue is a carboxy-terminal proline, which would add a third potential P-x-x-P motif.

Whether these changes affect the function of p13 and contribute to the distinct pathogenic properties of the HTLV-1C clade found in indigenous Australian patients [83] should be a point of investigation in future studies.

## Conclusions

Many studies have shed light on the expression, intracellular trafficking, and function of p13, but the function of this protein in the context of the complete viral genome remains elusive. Given the importance of mitochondria in anti-viral innate immunity, it will also be important to discover if, by targeting mitochondria, p13 may hamper the host cell’s anti-viral responses. Notably, several other human tumor viruses also code for mitochondrial proteins, with diverse effects on mitochondrial function and the viral life cycle [56].

In vivo study of a molecular clone of HTLV-1 without changing the start codon of HBZ is essential for determining the true importance of p13 in HTLV-1 infection. Galli et al. showed the possibility of utilizing humanized mice for studying the p12 protein of HTLV-1 in the whole virus using infected primary CD4^+^ cells [86]. This model may be useful for better assessing the pathogenic potential of p13 and investigating the intracellular localization of the protein in different tissues in infected animals in vivo. The identification of new roles for mitochondria opens up new questions about the role of p13 in both the pathogenesis of HTLV-1 and in immune responses. HTLV-1C involves up to half of some communities in endemic foci in Australia. The remarkable concentration of this virus demands that the functional role of p13 be better understood, and presents an opportunity to investigate the effects of the different sequence variations in the functional domains of p13.

## Data Availability

All data generated or analyzed during this study are included in published articles cited in the review and/or are available in GenBank https://www.ncbi.nlm.nih.gov/genbank/.

## Abbreviations

ATL: Adult T cell leukemia
BLAST: Basic local alignment search tool
CSFV: Classical swine fever virus
CVB: Coxsackie B virus
cGAS: Cyclic GMP–AMP synthase
ER: Endoplasmic reticulum
ETC: Electron transport chain
FPase: Farnesyl pyrophosphate synthase
GFP: Green fluorescent protein
HA: Hemagglutinin
HBZ: HTLV-1 bZIP factor
HIV: Human immunodeficiency virus
HSP: Heat shock protein
HTLV: Human T cell leukemia virus
HAM/TSP: HTLV-1 associated myelopathy/tropical spastic paraparesis
LTR: Long terminal repeat
MAVS: Mitochondrial antiviral-signaling protein
mtDNA: mitochondrial DNA
MTS: Mitochondrial targeting signal
NLS: Nuclear localization sequence
NFAT: nuclear factor of activated T cells
*orf*: Open reading frame
PTLV: Primate T cell leukemia virus
PTP: Permeability transition pore
ROS: Reactive oxygen species
SFK: Src family kinase
SH3: Src homology 3
STING: Stimulator of interferon genes
STLV: Simian T cell leukemia virus
T-ALL: T cell acute lymphoblastic leukemia
WB: Western blot
